# Evaluating the Feasibility and Acceptability of an Artificial-Intelligence-Enabled and Speech-Based Distress Screening Mobile App for Adolescents and Young Adults Diagnosed with Cancer: A Study Protocol

**DOI:** 10.3390/cancers14040914

**Published:** 2022-02-12

**Authors:** Anao Zhang, Aarti Kamat, Chiara Acquati, Michael Aratow, Johnny S. Kim, Adam S. DuVall, Emily Walling

**Affiliations:** 1 School of Social Work, University of Michigan, Ann Arbor, MI 48109, USA; 2 Adolescent and Young Adult Oncology Program, University of Michigan Health, Ann Arbor, MI 48109, USA; aartika@med.umich.edu; 3 Department of Pediatrics, University of Michigan Health, Ann Arbor, MI 48109, USA; 4 Graduate College of Social Work, University of Houston, Houston, TX 77204, USA; cacquati@central.uh.edu; 5 Department of Health Disparities Research, The University of Texas MD Anderson Cancer Center, Houston, TX 77030, USA; 6 Ellipsis Health, San Francisco, CA 94102, USA; mike@ellipsishealth.com; 7 Graduate School of Social Work, University of Denver, Denver, CO 80208, USA; johnny.kim@du.edu; 8 Department of Medicine, University of Chicago, Chicago, IL 60637, USA; duvalla@medicine.bsd.uchicago.edu

**Keywords:** adolescent and young adult cancer, distress, artificial intelligence, feasibility, acceptability, vocal biomarkers, voice analysis

## Abstract

**Simple Summary:**

Adolescent and young adult (AYA) patients diagnosed with cancer are at a higher risk of psychological distress, which requires regular monitoring throughout their cancer journeys. Paper-and-pencil or digital surveys for psychological stress are often cumbersome to complete during a patient’s visit, and many patients find completing the same survey multiple times repetitive and boring. Recent advances in mobile technology and speech science have enabled flexible and engaging ways of monitoring psychological distress. This paper describes the scientific process we will use to evaluate an artificial intelligence (AI)-enabled mobile app to monitor depression and anxiety among AYAs diagnosed with cancer.

**Abstract:**

Adolescents and young adults (AYAs) diagnosed with cancer are an age-defined population, with studies reporting up to 45% of the population experiencing psychological distress. Although it is essential to screen and monitor for psychological distress throughout AYAs’ cancer journeys, many cancer centers fail to effectively implement distress screening protocols largely due to busy clinical workflow and survey fatigue. Recent advances in mobile technology and speech science have enabled flexible and engaging methods to monitor psychological distress. However, patient-centered research focusing on these methods’ feasibility and acceptability remains lacking. Therefore, in this project, we aim to evaluate the feasibility and acceptability of an artificial intelligence (AI)-enabled and speech-based mobile application to monitor psychological distress among AYAs diagnosed with cancer. We use a single-arm prospective cohort design with a stratified sampling strategy. We aim to recruit 60 AYAs diagnosed with cancer and to monitor their psychological distress using an AI-enabled speech-based distress monitoring tool over a 6 month period. The primary feasibility endpoint of this study is defined by the number of participants completing four out of six monthly distress assessments, and the acceptability endpoint is defined both quantitatively using the acceptability of intervention measure and qualitatively using semi-structured interviews.

## 1. Introduction

Adolescents and young adults (AYAs) diagnosed with cancer are an age-defined patient population between 15 and 39 years old, with an estimated global incident rate of 1.2 million new diagnoses in 2019 [1]. In the United States, there were approximately 90,000 newly diagnosed AYA cancer patients and a total of 630,000 survivors of AYA cancer in 2020 [2,3]. AYA cancer survivors, including those receiving active treatment and post-treatment survivorship care, suffer distinct and disproportionately more adverse outcomes than their pediatric and older adult counterparts [4]. These challenges include disruptions in school or work, financial toxicity, disruption in psychosocial development, and oncofertility, just to name a few [5,6,7]. Notably, these challenges have significantly compromised AYA cancer survivors’ psychological wellness, including the fear of death and recurrence [8], grief due to loss of reproductive capabilities and potential for having a family in the future [9], forced dependence upon others [10], and most importantly, psychological distress, including depression and anxiety [11,12].

Psychological distress is highly prevalent among AYA cancer survivors, as evidenced by studies reporting a 45% or higher prevalence rate of clinically significant distress symptoms requiring intervention [13,14]. Unaddressed psychological distress among AYA cancer survivors is associated with poor disease management, low quality of life, and increased suicide risk [15,16]. Most AYA cancer survivors, however, do not receive timely and appropriate treatment for psychological distress due to inadequate screening and monitoring of depressive and anxiety-related symptoms. While the American College of Surgeons’ Commission on Cancer (CoC) has mandated compliance with distress screening as a condition for accreditation [17], routine distress screening protocols remain poorly implemented, with only one in three cancer patients being screened as per the protocols [18,19]. Alarmingly, comprehensive cancer centers, such as the National Cancer Institute (NCI)-designated centers in the United States, have the lowest screening rate in comparison to other cancer care facilities, and rates of AYA cancer survivors’ screening for distress are significantly lower compared to those of their adult counterparts [19].

Three key factors contribute to poor distress screening and monitoring among AYA cancer survivors. First, oncologists and other providers do not have time and often lack the expertise to accurately assess and intervene for psychological distress. For example, 40% of oncologists find routine distress screening impractical and 56% of providers are unsure of the clinical interpretation of distress measures [20,21]. Second, AYA cancer survivors are the least likely group to disclose symptoms of psychological distress in comparison to adult patients due to emotional burden or by their desire not to worry family members [22,23]. Studies have consistently documented the emotional burden and maladaptive coping behaviors among AYA cancer survivors experiencing psychological distress, such as self-isolation and social withdrawal [24]. Finally, even for cancer centers that successfully implement distress screening protocols, most assessments are one-time efforts and do not longitudinally monitor the trajectory of psychological distress. This is problematic because AYA cancer survivors’ distress trajectories fluctuate over time, and it is critical to monitor patients’ distress throughout their cancer journey [25,26], especially during pivotal treatment time points.

Studies have documented four pivotal time points when the risk of clinical distress peaks throughout a patient’s cancer journey: 2–4 weeks after a new diagnosis, within 2 weeks of starting or changing to new treatment (e.g., chemotherapy to bone marrow transplant), during the transition from active treatment with a curative intention to post-treatment survivorship care (2–4 weeks after finishing treatment), and one year post-treatment [25,27]. Taken together, it is crucial to create and evaluate innovative methods that are flexible, engaging, and effective in monitoring psychological distress among AYA cancer survivors over time. Recent advances in speech-based distress assessment and AI have provided promising solutions to the aforementioned challenges.

### 1.1. Speech-Based Distress Assessment Tools

Speech-based distress assessment tools that are automatic or AI-based have advanced remarkably over the past decade, especially those using voice acoustics to screen for depression and anxiety [28,29,30]. In comparison to psychometrically validated scales of depression and anxiety, speech-based assessment tools not only have comparable diagnostic performance [31,32] but also clear advantages, including (1) their unobtrusiveness, which reduces the patient burden experienced with manually completed scales; (2) the use of speech data (including voice biomarkers), which minimizes human error during scale administration such as missing value or outliers; and (3) the cathartic nature of talking/venting, which reduces patients’ fatigue due to completing the survey repeatedly [33,34,35].

Despite these advancements, several gaps remain in the speech-based distress assessment literature. First, most existing tools only use one type of speech data, either acoustic (the sound of the voice) or semantic (the words used) data, but not both [36,37]. This has limited the use of different types of speech data to improve assessment precision. Second, a recent systematic review found that no tool so far was equipped to evaluate D&A simultaneously. About half of the studies on speech-based mental health assessment focused on depression, but only a small number (5%) examined anxiety, whereas studies of both conditions are rare [36,38]. It is problematic because depression and anxiety often co-occur, and anxiety disorders are highly prevalent among AYA survivors, e.g., health anxiety or fear of reoccurrence. Finally, to the best of our knowledge, none of the existing tools offer an oncologist-facing dashboard that provides easy, comprehensive, and informative access to patients’ D&A data [35,36]. This is a missed opportunity to close the screening-to-intervention gap, to streamline the care process, and to enable early detection and timely intervention for psychological distress among AYA survivors.

In this study, we propose to evaluate a state-of-the-art speech-based D&A assessment tool, the Ellipsis Health Voice Tool (EH Voice Tool), that has the potential to address all the aforementioned gaps. The proposed platform uses artificial intelligence (AI), which integrates a patients’ acoustic and semantic data and simultaneously produces assessment results for both depression and anxiety. In addition, the platform offers an oncologist-facing dashboard, which facilitates patient referral for psycho-oncology services and allows timely coordination of care and patient-/person-centered approaches. Finally, Ellipsis Health has published a series of peer-reviewed technical papers validating the machine learning algorithms as well as the speech recognition performance that power the approach [39,40,41,42,43].

### 1.2. The Ellipsis Health Voice Tool (EH Voice Tool)

The EH Voice Tool is a mobile app that connects to a cloud-based AI system that analyzes patients’ speech data obtained through 90 s “conversations” on selected topics, including some related to cancer. The EH Voice Tool’s D&A risk prediction models perform well across demographic classes, including self-reported gender, age, and race/ethnicity. Unlike other existing tools using feature extraction of isolated semantic or acoustic models for prediction, the EH Voice Tool integrates both semantic and acoustic information via its AI-enabled algorithms. Utilizing both acoustic and language modeling is achieved through modern deep learning architectures and transfer learning. Unlike older acoustic modeling approaches for this domain, the Ellipsis acoustic system directly models the signal. This modeling strategy results in far better performance than other approaches that rely on predetermined features such as pitch or energy. Overall, these methods enable both the language and acoustic models to continuously improve as more training data become available.

The models that underlie the EH Voice Tool show top performance in comparison to results published in the literature [44]. Specifically, results for the EH Voice Tool’s performance on PHQ/GAD prediction are currently 0.85/0.84 for area under the curve (AUC; for binary classification with a threshold of 10), 4.25/4.47 for root mean square error (RMSE, a measure of average regression error), and 3.13/3.23 for mean absolute error (MAE, another measure of average regression error) [38,40]. Finally, to the best of our knowledge, the EH Voice Tool is the only speech-based distress screening and monitoring tool that simultaneously generates results for depression and anxiety scores, an essential strategy for reducing fatigue over long-term and repetitive screening.

**Figure 1:**
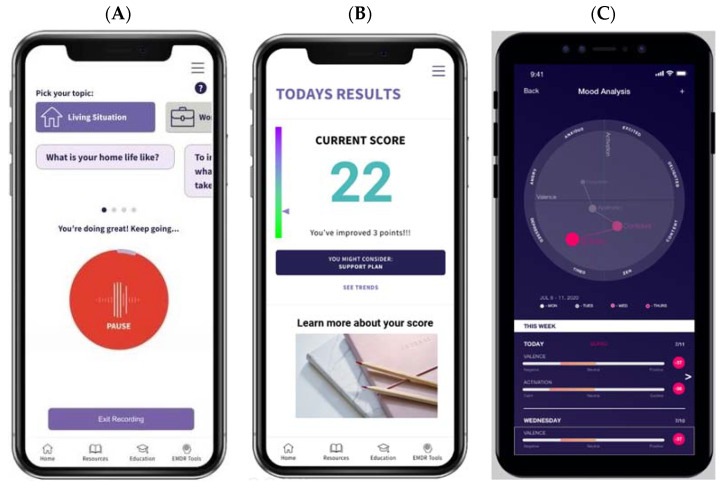

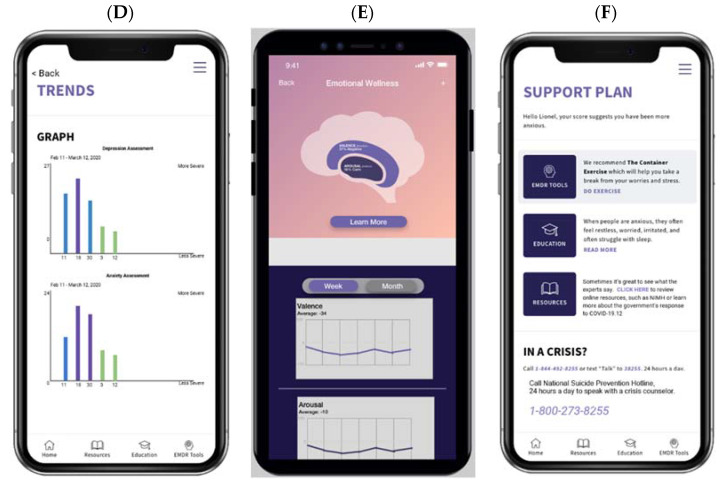
EH Voice Tool mobile app user interface. (**A**) voice collection, (**B**) numeric results, (**C**) visual results, (**D**) trend over time option 1, (**E**) trend over time option 2, (**F**) resource center.

The EH Voice Tool can be easily downloaded to Apple and Android devices. Figure 1A–F presents the EH Voice Tool’s user interface. After login and selecting “start”, a patient can “talk to” the app about various topics, e.g., health, work, or life, for 90 s (Figure 1A). After each recording, patients see a numeric score and a visual interpretation of their assessment results usually within one minute of completion. If patients wish to learn more about their scores, they have the option to do so (Figure 1B,C). Then, they are taken to the next page, where historical trends of D&A are presented (Figure 1D,E). Patients also have access to a mental health resource center (Figure 1F) where guided meditations, psycho-educational resources, and information about the national crisis hotline for suicide are available. Different color themes and styles are available to personalize the interface. The EH Voice Tool version to be evaluated has already incorporated feedback received from AYAs with and without cancer on the conversational topics used to collect patients’ speech data. Finally, EH Voice Tool has a web-based HIPPA-compliant dashboard portal outlining all patients’ distress trajectories for their respective treating oncologists, which streamlines the assessment with the intervention care continuum.

## 2. Materials and Methods

### 2.1. Study Design

This single-group/arm prospective cohort study will employ a mixed-methods approach, including (1) tracking a cohort of 60 AYA cancer survivors’ psychological distress (i.e., D&A) over 6 months; (2) documenting AYA cancer survivors’ utility of the tool and describing the trajectory of their psychological distress over time; (3) conducting semi-structured qualitative interviews with a subsample of study participants and with oncologists.

### 2.2. Inclusion Criteria

This study’s inclusion criteria are: (1) having or had a cancer diagnosis; (2) between the ages of 15 to 26 years old at the time of enrollment; (3) willing to provide informed consent (and assent if applicable); and (4) native speaker of English or highly fluent. Exclusion criteria are: (1) having active psychosis; (2) having a suicidal risk at an elevated level, i.e., ideation with an active plan or more severe; (3) currently receiving end-of-life care; or (4) having cancers that affect speaking. Unlike NCI’s definition of AYA between 15 to 39 years old, this study only focuses on those between 15 to 26 years because adolescence/emerging adulthood (15 to 26 years) is a distinctly different developmental stage from young adulthood (27 to 39 years) [45]. Therefore, AYA cancer patients from the two developmental stages have unique psychosocial challenges and are most likely to have different distress trajectories and need to be studied separately. We will use a stratified sampling approach with the patient’s age, sex, and the pivotal treatment timepoints (defined earlier) being the stratifying factors (Table 1).

### 2.3. Participant Recruitment and Study Procedures

AYA cancer survivors will be recruited at the University of Michigan Rogel Cancer Center, C.S. Mott Children’s Hospital Division of Hematology/Oncology, and the Michigan Medicine AYA Oncology Program. Recruitment will occur over 12 months to maximize sample size. Two trained research staff (RS) will visit the outpatient and inpatient clinics at C.S. Mott Children’s Hospital Division of Hematology/Oncology and the Sarcoma Clinic at Rogel Cancer Center when the patients are waiting for their appointments. We will also work closely with the patient navigator of the Michigan Medicine AYA Oncology Program to promote study recruitment and enrollment. In addition to active recruitment, study investigators (A.K. and E.W.) will share the study with their colleague pediatric oncologists and encourage them to refer participants to the study.

Upon successful recruitment, a trained RS will schedule an initial meeting (T0) to obtain consent/assent and conduct a baseline assessment. The RS will also provide instructions for using EH Voice Tool. A participant will be asked to use EH Voice Tool once a month for 6 months, i.e., a total of 6 times (T1–T6). Participants will receive phone-based notifications once a month reminding them about using the mobile app. If a participant misses a scheduled assessment, the mobile app will send two more notifications 24 and 48 h after. Participants adherence to the EH Voice Tool will be monitored using the platform administrative data of each participant’s usage of the tool, e.g., how many times a participant used the app and for how long each time. By the end of month 6, the RS will meet with all participants for a brief post-study assessment (T7). Table 2 describes all measures and the time of measure administration for the study. Finally, the RS will reach out to a subsample of participants (*n* = 15), including 10 participants who achieve the feasibility endpoint (defined later) and 5 who do not achieve the feasibility endpoint and conduct semi-structured qualitative interviews to evaluate participants’ perceived feasibility and acceptability of the EH Voice Tool, and identify facilitators and barriers associated with EH Voice Tool uptake.

The investigative team will also utilize the dashboard feature of the EH Voice Tool and provide an overview of AYA cancer patients’ D&A trajectory for oncologists whose patients participate in this study. Specifically, we will email each participating oncologist monthly a pdf document summarizing their patients’ D&A scores. In addition, each oncologist can log in to their provider-specific dashboard (an HIPPA-compliant web portal) to obtain more details about their monthly summary, e.g., trends in a specific patient’s D&A score or if any referral is made. We will interview 10 oncologists by the end of the study to evaluate their perceived feasibility and acceptability and identify facilitators and barriers associated with EH Voice Tool uptake.

**Participant safety.** We will closely monitor the EH Voice Tool backstage data to ensure patient safety. No specific action will be taken if a participant scores mild or lower depression and/or anxiety. If a participant scores moderate or moderately severe depression and/or anxiety, we will immediately notify the clinic social worker to provide follow-up services and the research staff will follow up with the participant within 48 h. If a participant scores severe depression and/or anxiety, a research staff will immediately conduct a safety check-in with the participant and notify the clinic social worker to follow up.

### 2.4. Study Endpoints

The primary feasibility endpoint will be measured by the observed number of times each participant completes the EH Voice Tool out of the total possible times completing the tool, i.e., 6 times. If a participant completes the EH Voice Tool 4 or more times during a 6 month period, i.e., ≥66.66% rate of completion, that participant is regarded as achieving the feasibility endpoint. This feasibility study will be considered as successful if 60% or more of the participants, i.e., ≥36, reach the feasibility endpoint. The 60% benchmark for success was selected based on a national study reporting a 53% completion rate of one-time distress screening among U.S. cancer patients [18]. Therefore, we consider a 60% multiple-time (versus one-time) distress monitoring compliance rate being superior to the current practice of distress screening.

The secondary acceptability endpoint will be measured by the Acceptability of Intervention Measure, which is a psychometrically validated implementation scale that specifically measures treatment acceptability. An average score of 4.0/5.0 will be considered as evidence of EH Voice Tool acceptability.

We will also collect demographic information of all participants via self-report, which will include patient age, sex, gender identity, and race/ethnicity. Clinical information abstracted directly from patient electronic health records (EHRs) will include cancer diagnosis, prescribed therapy, and staging of disease. During the baseline assessment, we will evaluate a participant’s general mental status using the MINI interview; depression using the Patient Health Questionnaire, 8 items (PHQ-8); and anxiety using the Generalized Anxiety Disorder, 7 items (GAD-7).

The PHQ-8 is an 8-item valid depression measure both for the general population and for individuals with cancer [46]. The PHQ-8 asks questions about patients’ common symptoms of depression over the past two weeks, e.g., feeling tired or having little energy, poor appetite, or overeating, and has been widely recommended as a measurement option for major depressive disorders both in the current and previous versions of the Diagnostic and Statistical Manual of Mental Disorders, i.e., DSM-IV-TR and DSM-5 [47,48]. The excellent psychometric property of the PHQ-8 has been reported across different age and racial/ethnic groups, endorsing its utility among cancer patients in the United States [49,50,51]. The GAD-7 is a valid, 7-item anxiety measure both for the general population and for individuals with cancer [52,53]. The GAD-7 inquires about patients’ common symptoms of anxiety or anxious mood over the past two weeks, such as feeling nervous, anxious or on edge, or trouble relaxing. The GAD-7 has been supported with strong psychometric properties across age groups and racial/ethnic backgrounds, including those diagnosed with cancer [54,55,56].

### 2.5. Data Analysis

Descriptive statistics of participants’ demographic information and measures will be conducted. We will summarize the statistic of the number of study participants who achieve the feasibility endpoint over the total study sample size (N = 60), i.e., a percentage statistic. Any study participant who completes at least 4 out of 6 assessments during the study will be considered as reaching the feasibility endpoint. It is possible that a study participant may complete the EH Voice Tool more frequently than designed, e.g., completing EH Voice Tool more than once per month, leading to a total number of assessments greater than 6 times over the 6 month study period. These participants will be viewed the same as those who complete 4–6 assessments during the study period. In addition, we will also report descriptive statistics for the Acceptability of Intervention Measure, i.e., mean and standard deviation, to quantify the participants’ perceived EH Voice Tool acceptability.

We will also explore if AYA cancer patients’ D&A scores (measured via the EH Voice Tool) differ by a patient’s current stage of pivotal timepoint, i.e., 2–4 weeks after a new diagnosis, within 2 weeks of (new) treatment (including changes in planned treatment), during the transition to survivorship care (2–4 weeks finishing treatment), and one year after the completion of treatment. For this analysis, we will conduct a one-way between group analysis of variance (ANOVA) with the PHQ-8 and GAD-7 at each timepoint as the (continuous) dependent variable and the 4 pivotal treatment timepoints as the grouping variable. In addition, we will also explore if the change in the PHQ-8 and GAD-7 scores over time, i.e., over the 6 times, differ by the 4 pivotal timepoints through latent growth curve modeling. We understand that our planned sample size of 60 participants may not provide sufficient power and regard this analysis only as exploratory.

Qualitative interview data with AYA cancer patients (*n* = 15) and oncologists (*n* = 10) will first be transcribed verbatim and coded using thematic content analysis in NVivo 12 (QSR International Pty Ltd., 2018, https://www.qsrinternational.com/nvivo-qualitative-data-analysis-software/home, accessed on 16 December 2021), with discrepancies resolved through discussion. Axial code mapping will organize data into higher categories of themes related to the EH Voice Tool’s acceptability: (1) participants’ and oncologists’ perceived acceptability, (2) participants’ and oncologists’ perceived strengths and weaknesses of EH Voice Tool, (3) participants’ and oncologists’ perceived facilitators of EH Voice Tool uptake, and (4) participants’ and oncologists’ perceived barriers of EH Voice Tool.

### 2.6. Power Calculation and Sample Size Considerations

As defined earlier, the feasibility endpoint is defined as a participant completing at least 4 out of 6 assessments throughout the study period; and the primary objective of this study will be successful if at least 60%, i.e., 36 out of 60, achieve this endpoint by the end of the study. If successful, the observed completion rate will be at least 36 out of 60, which corresponds to a one-sided Wilson’s 95% confidence interval equal to (0.473, 0.714).

## 3. Discussion

This single-group/arm prospective cohort study, to the best of our knowledge, will be among the first to evaluate the use of artificial intelligence and speech data via a mobile app to facilitate distress screening and monitoring for AYAs diagnosed with cancer. It is well-established that distress monitoring is essential to quality cancer care for AYAs diagnosed with cancer, yet is often difficult to achieve in real-world practices [57,58]. Therefore, it is vital to identify alternative innovative distress monitoring methods to traditional distress screening practices, e.g., distress thermometer or paper-and-pencil/digital surveys. This is especially important for the AYA cancer population as they are most likely to disengage from existing distress screening methods [59]. A mobile app that extracts cancer survivors’ speech data has significant potential to offer a flexible, attractive, and developmentally appealing way to implement distress monitoring among the AYA population. Therefore, the findings of this pilot study will provide invaluable information regarding patient and provider-reported feasibility and acceptability data of the tool. With the preliminary data supporting the clinical utility of the tool, we will be able to further evaluate and confirm the efficacy of the EH Voice Tool in a larger population of patients, such as in a multicenter, randomized, controlled trial study to obtain definitive evidence supporting the EH Voice Tool’s application in AYAs diagnosed with cancer.

We will analyze qualitative feedback from both AYA cancer survivors and oncologists treating AYA cancer patients to further improve the EH Voice Tool’s clinical utility and helpfulness. We consider it a notable strength of the proposed study that we will receive qualitative feedback from both patients and the oncologists so that the tool not only connects with AYA cancer survivors but is also perceived as helpful by providers. The investigative team will then utilize the preliminary findings from this study to inform a larger and more rigorous trial, i.e., a multicenter, randomized, controlled trial, to further evaluate the tool’s clinical efficacy in identifying and monitoring distress trajectories among AYA cancer survivors in comparison to existing distress screening protocols, e.g., distress thermometer or electronically administered distress measures. We think that given the ubiquity of mobile app usage among the AYA age group, the EH Voice Tool will demonstrate superiority over existing distress screening protocols.

Finally, psychological distress, i.e., depression and anxiety, are salient factors impacting AYA cancer survivors’ quality of life [60,61]. Through the early detection and ongoing monitoring of psychological distress via the EH Voice Tool, another important direction of future research is to evaluate if delivering the EH Voice Tool will impact AYA cancer survivors’ long-term quality of life and other domains of patient-reported outcomes.

## 4. Conclusions

Mobile-app-based tools that integrate artificial intelligence and patients’ speech data possess compelling advantages in monitoring psychological distress among individuals diagnosed with cancer, especially for the AYA population—a tech-savvy generation. This pilot study has the potential to revolutionize distress screening practices for AYA cancer survivors and, more broadly, psycho-oncology communities. Taking advantage of the latest advancements in information technology and distress diagnostics, the proposed study will streamline the distress monitoring process for AYAs diagnosed with cancer.

## Figures and Tables

**Table 1:** Clustered recruitment strategy.

Recruitment Clusters	ND	CT	ET	PT	Total
Male Adolescent (15–17 years)	4	4	4	4	16
Female Adolescent (15–17 years)	4	4	4	4	16
Male emerging adult (18–26 years)	4	4	3	3	14
Female emerging adult (18–26 years)	4	4	3	3	14
Total	16	16	14	14	60

ND = newly diagnosed; CT = change in treatment; ET = end of therapy; PT = one-year post treatment.

**Table 2:** Study measures and assessment time points *.

Measure	Measure Administration Time Points and Personnel			
	T0	T1–T6	T7	Admin
MINI Interview	X			RS
PHQ-8	X			RS
GAD-7	X			RS
EH Voice Tool Admin. Data		Ongoing all participants		Platform
AIM Measure			X	RS
Qualitative Interview			X	RS

* MINI Interview = Mini-International Neuropsychiatric Interview; PHQ-8 = Patient Health Questionnaire, 8-item; GAD-7 = Generalized Anxiety Disorder, 7-item; EH Voice Tool Admin. Data = EH Voice Tool Back Stage Administrative Data; AIM Measure = Acceptability of Intervention Measure; RS = research staff; Platform = platform-based data administration.

## Data Availability

The data presented in this study are available in the main text.
